# Subcutaneous administration of isatuximab in patients with multiple myeloma by an on-body delivery system: results of a nurse survey

**DOI:** 10.3389/fonc.2025.1547108

**Published:** 2025-06-18

**Authors:** Nuria Sánchez Avello, Paula Calvo Pajares, Paul Cordero, Florence Suzan, Connie Barlas

**Affiliations:** ^1^ Hospital Universitario Marqués de Valdecilla (IDIVAL), Santander, Spain; ^2^ Sanofi Research & Development, Reading, United Kingdom; ^3^ Sanofi Research & Development, Vitry-sur-Seine, France; ^4^ Epworth HealthCare, Melbourne, VIC, Australia

**Keywords:** multiple myeloma, isatuximab, administration, subcutaneous, on-body delivery system, nurse, survey

## Abstract

**Introduction:**

Subcutaneous (SC) administration of the anti-CD38 antibody isatuximab (Isa) by an on-body delivery system (OBDS), plus pomalidomide-dexamethasone, has demonstrated safety and efficacy comparable to intravenous (IV) administration, with no infusion reactions and excellent local tolerability in multiple myeloma (MM) patients. We report here results of a nurse survey designed to evaluate convenience of treatment with SC Isa, via OBDS, for healthcare providers and MM patients.

**Methods:**

A newly developed, expert-vetted questionnaire was used to survey nurses with experience in SC administration to MM patients enrolled in clinical trials. Results on extent of agreement with pre-vetted statements were expressed as percentages of respondents. Free-text answers were analyzed for each respondent and grouped by topic.

**Results:**

All surveyed nurses (N=12) agreed that OBDS administration improved efficiency and was easy to learn and administer with a low level of physical burden, leading to a preference for OBDS over IV Isa administration and facilitating a positive treatment experience for the patients. Compared with IV dosing, the OBDS improved patient comfort and could reduce time spent in the clinic. As agreed by most nurses, main advantages for patients included no needle visibility, short treatment duration, and a generally well-tolerated and painless SC injection.

**Conclusions:**

Our findings show a high level of confidence among nurses in SC Isa administration via OBDS, due to the ease of use, tolerability, and time savings achieved with hands-free OBDS injections. Our findings suggest applicability of the OBDS for convenient SC Isa administration to MM patients in routine clinical practice.

## Introduction

Findings from multiple clinical trials have demonstrated that addition of the anti-CD38 monoclonal antibody isatuximab (Isa) to standard regimens can provide benefit to patients across the therapeutic spectrum for multiple myeloma (MM), in quadruplet combinations for newly diagnosed MM (NDMM) and in triplet combinations for relapsed/refractory MM (RRMM), with a manageable safety profile (1–11). Isa is currently approved in many countries for the treatment of patients with RRMM by IV administration, in combination with pomalidomide and dexamethasone (Pd) after two or more therapies, and with carfilzomib and dexamethasone after one prior therapy (5, 6, 12, 13). Based on the results of the Phase 3 IMROZ trial (NCT03319667), IV Isa has been recently approved in the United States in combination with bortezomib, lenalidomide, and dexamethasone for the treatment of patients with newly diagnosed MM not eligible for an autologous stem cell transplant (8, 12).

To facilitate treatment with Isa and offer more convenience to patients and healthcare providers (14–17), subcutaneous (SC) administration of Isa by an infusion pump or an investigational on-body delivery device (OBDS, **Supplementary Figure S1**) was evaluated in a multi-center, multi-cohort Phase 1b study (NCT04045795) in patients with RRMM who had received two or more prior treatment lines, compared to IV infusion (18). Results from this study showed safety and efficacy of treatment with SC Isa in combination with Pd comparable to those observed with IV Isa plus Pd in the ICARIA-MM study (18). In addition, SC administration of Isa via OBDS in RRMM patients enrolled in the expansion cohort of the study demonstrated excellent local tolerability with no infusion/injection reactions (IRs) nor treatment interruptions in any of the patients receiving SC Isa by OBDS at the recommended Phase 2 dose of 1400 mg. Injection site reactions (grade 1; occurring in 1.7% of all OBDS administrations), consisted mostly of erythemas. The overall response rate was 66.7% in the IV cohort and 72.7% in the OBDS cohort (18). SC Isa is being further evaluated in randomized Phase 3 studies in patients with NDMM (GMMG-HD8/DSMM XIX trial; NCT05804032) and RRMM (IRAKLIA trial; NCT05405166) as well as in Phase 2 studies in RRMM (19–22).

In order to evaluate SC Isa treatment delivery from the perspective of healthcare professionals (HCPs), as an approach to help minimize time in the clinic and decrease patient burden, we conducted an online survey among nurses who had acquired clinical experience with SC Isa administration in the Phase 1b study and in the randomized Phase 3 GMMG-HD8/DSMM XIX study. In the latter, ongoing trial, SC Isa is being investigated in combination with lenalidomide, bortezomib, and dexamethasone for the treatment of transplant-eligible patients with NDMM (19).

We report here the results of this *ad hoc* nurse survey, which evaluated the treatment experience with SC Isa via OBDS for HCPs and for patients with RRMM or NDMM, by exploring multiple aspects of the administration and monitoring processes from a nurse perspective.

## Materials and methods

### Survey questionnaire and analysis

Objectives of this survey were to collect and analyze feedback from clinical trial nurses on their experiences with SC Isa administration, using a newly created, online questionnaire. The nurses and the investigators who contributed to development of the questionnaire had expertise in SC administration of monoclonal antibodies to cancer patients, clinical research, patient reported outcomes, and the device. The 32 survey questions were designed to assess the nurses’ experiences with SC Isa treatment administration via OBDS, IV Isa infusion, and SC administration (by manual push) of large volume (15-mL) antibody formulations (eg, daratumumab), as well as the patient experiences with the OBDS as perceived from the nurses’ perspective.

Most questions were provided as statements; nurses would indicate their level of agreement via a 5-point Likert scale with 5 = “I completely agree”, 4 = “I agree, 3 = “neither agree, nor disagree” (neutral), 2 = “I don’t agree”, and 1 = “I do not agree at all”. For analysis and presentation, 4 and 5 ratings were pooled as “agree/completely agree” and 2 and 1 ratings as “do not agree/do not agree at all”. The participating nurses were also invited to answer questions with other modalities (eg, multiple-choice answers) or provide unaided, free-text answers for selected questions, to further explore their experience with administration of SC treatment and the related patient management.

After completion of the online survey, results were combined for all the anonymized respondents and expressed as percentages of respondents with mean and median ratings when applicable. Free-text answers were analyzed for each respondent and grouped by main topic addressed.

### Survey participants

Registered nurses, who participated in the Phase 1b study of SC Isa (NCT04045795) (18) and the Phase 3 GMMG-HD8/DSMM XIX (NCT05804032) trial (19) were invited to complete the online survey questionnaire in their native languages (English, French, and German), from December 8, 2023 to January 3, 2024. They were located at clinical sites in Australia, France, Germany, and Spain.

### Patient treatment in clinical trials

In the multi-center, open-label Phase 1b study, patients with RRMM received IV Isa at 10 mg/kg or SC Isa (in the expansion cohort) via an investigational injector device, OBDS, manufactured by Enable Injections, Inc. (Cincinnati, OH, USA), at the recommended Phase 2 dose (RP2D) of 1400 mg (18). Isa was administered SC via OBDS at a single injection site on the abdomen that was rotated at each administration. IV and SC Isa were given once weekly for 4 weeks in cycle 1 and then once every 2 weeks in the subsequent 28-day cycles, in combination with standard doses of pomalidomide and dexamethasone (5). In most instances, the clinical trial nurses received the OBDS prefilled with Isa by the hospital pharmacy. Patients enrolled in this study had RRMM and had received 2 or more prior lines of MM treatment (18).

In the multi-center, randomized, Phase 3 GMMG-HD8/DSMM XIX study, transplant-eligible patients received 3 cycles of IV Isa in arm A or 3 cycles of SC Isa in arm B in the induction phase, in combination with lenalidomide, bortezomib, and dexamethasone, followed by standard intensification (e.g. cyclophosphamide-based mobilization therapy, stem cell collection, and high-dose melphalan), and autologous stem cell transplantation (ASCT). IV Isa at 10 mg/kg and SC Isa at a dose of 1400 mg were administered weekly in the first cycle (on days 1, 8, 15, 22, and 29), and biweekly in the 2 subsequent cycles (on days 1, 15, and 29). Patients on this trial had a confirmed diagnosis of untreated NDMM requiring systemic therapy, and were eligible for high-dose melphalan therapy and ASCT (19).

## Results

### Survey participants

All 12 nurses invited for the survey provided answers to the questionnaire. Ten of the 12 nurses had participated in the Phase 1b study and 2 nurses in the Phase 3 GMMG-HD8/DSMM XIX trial. Five of the nurses were at a clinical site located in France, 4 at a site in Spain, 2 at a site in Germany, and 1 in Australia.

Overall, 75% of the nurses (9/12) had experience with Isa administration via OBDS and all of them (12/12) with patient monitoring and patient education. All the nurses confirmed previous experience with IV infusion of antibody therapy and 83% of them (10/12) with SC administration (by manual push) of large-volume (15 mL) antibody formulations with a syringe.

### Nurse and patient experience with SC Isa administration via OBDS

**Figure 1:**
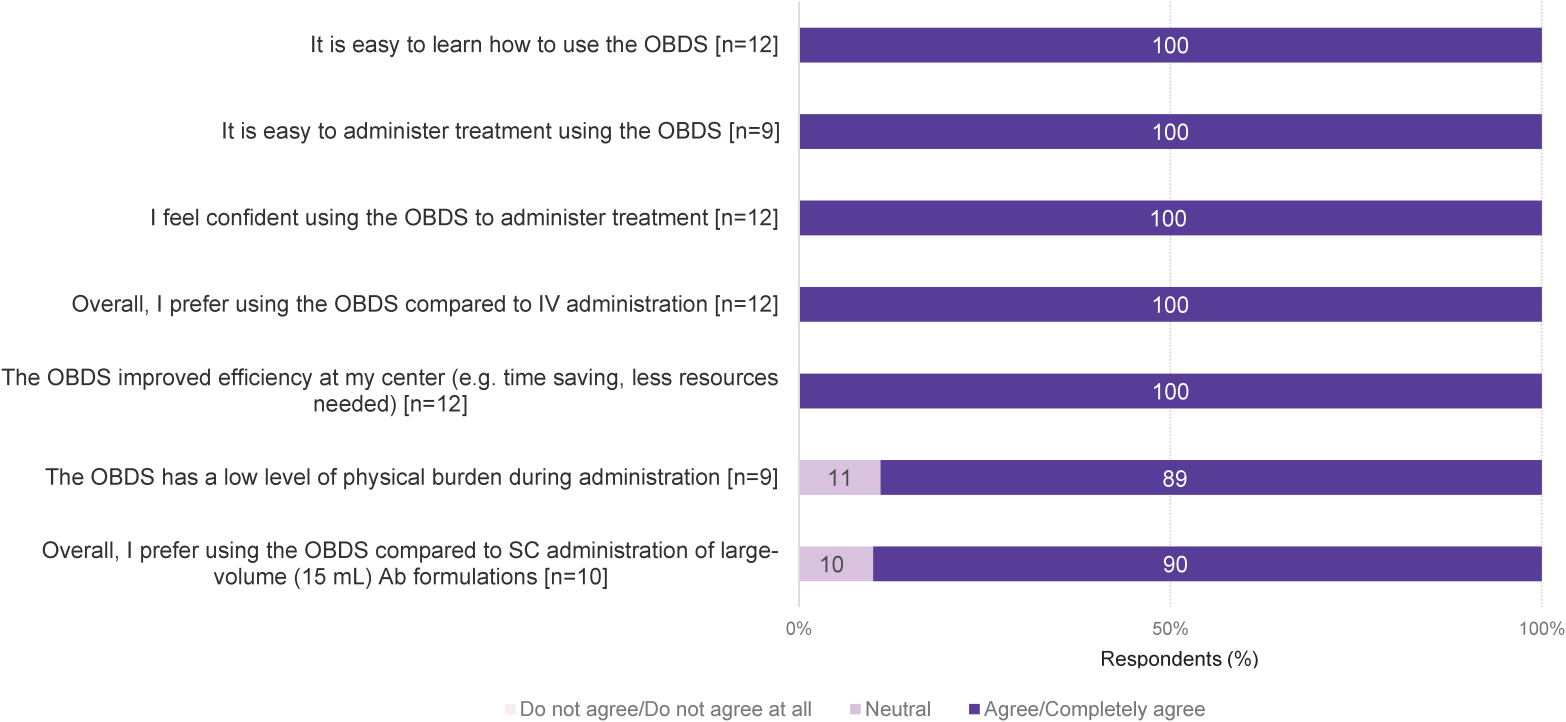
Overall nurse experience with SC Isa administration via OBDS. A 5-point Likert scale was used to indicate the level of agreement, where 5 meant “I completely agree” and 1 meant “I do not agree at all”. 4 and 5 ratings were pooled as “agree/completely agree” and 2 and 1 ratings as “do not agree/do not agree at all”. Ab, antibody; Isa, isatuximab; OBDS, on-body delivery system; SC, subcutaneous; IV, intravenous.

All nurses (12/12) felt confident using the OBDS to administer SC Isa treatment. They agreed that OBDS administration was easy to learn and to administer (100% of respondents; 12/12 and 9/9, respectively), improved efficiency (100%; 12/12), and had a low level of physical burden (89%; 8/9). The OBDS was preferred to IV dosing (100%; 12/12) and to SC administration (by manual push) of large-volume antibody formulations with a syringe (90%; 9/10) (**Figure 1**).

The main advantages identified for HCPs in using the OBDS for SC Isa in patients with MM were hands-free delivery, which allows small activities during administration or treatment of more than one patient with the OBDS (100% of respondents; 12/12), reduced physical burden compared to SC administration by manual push of large-volume (15 mL) antibody formulations (100%; 12/12), facilitated patient management (100%; 12/12), controlled delivery of treatment at a constant flow rate (92%; 11/12), no needle exposure (92%; 11/12), and labor saving (92%; 11/12) (**Table 1**). In addition, in the free-text answers, the nurses noted the ease of use, fast delivery/time savings, reduced risk of errors, and optimization of health care resources as advantages of the OBDS for HCPs (**Supplementary Table S1**).

**Table 1:** Assessment of SC Isa treatment administration via OBDS using a 5-point scale: advantages for HCPs.

Survey results	N	Mean rating	Median rating	Do not agree/do not agree at all (%)	Neutral (%)	Agree/completely agree (%)
Hands-free delivery of treatment, allowing small activities during administration or administering treatment via the OBDS to >1 patient	12	4.92	5.00	0	0	100
Reduced physical burden for HCPs compared to SC administration of large-volume (15 mL) antibody formulations with a syringe (requiring manual push of the syringe plunger)	12	4.75	5.00	0	0	100
Facilitated patient management	12	4.58	5.00	0	0	100
No needle exposure	12	4.75	5.00	0	8	92
Controlled delivery of treatment (constant flow rate)	12	4.67	5.00	0	8	92
Labor-saving	12	4.17	4.00	8	0	92

A 5-point Likert scale was used to indicate the level of agreement, where 5 meant “I completely agree” and 1 meant “I do not agree at all”. 4 and 5 ratings were pooled as “agree/completely agree” and 2 and 1 ratings as “do not agree/do not agree at all”. HCP, healthcare professional; Isa, isatuximab; OBDS, on-body delivery system; SC, subcutaneous.

83% of the nurses (10/12) agreed that “if an injection is interrupted, it is not clear how much drug has been administered” could be a potential limitation for HCPs, although no interruptions were observed in 581 administrations of SC Isa via OBDS in the Phase 1b study (**Table 2**) [18]. In the free-text answers, some nurses addressing potential negative perceptions mentioned the size/bulk of the device or package, the need of training and a learning curve, and lack of trust in automation of SC administration.

**Table 2:** Assessment of SC Isa treatment administration via OBDS using a 5-point scale: potential limitations for HCPs.

Survey results	N	Mean rating	Median rating	Do not agree/do not agree at all (%)	Neutral (%)	Agree/completely agree (%)
If the injection is interrupted, it is not clear how much drug has been administered	12	4.58	5.00	0	17	83
Plastic waste of this single-use device	12	4.08	4.00	0	33	67
Need to press and hold a button to pause the injection (active pause)	12	4.00	4.00	0	33	67

A 5-point Likert scale was used to indicate the level of agreement, where 5 meant “I completely agree” and 1 meant “I do not agree at all”. 4 and 5 ratings were pooled as “agree/completely agree” and 2 and 1 ratings as “do not agree/do not agree at all”. HCP, healthcare professional; Isa, isatuximab; OBDS, on-body delivery system; SC, subcutaneous.

**Figure 2:**
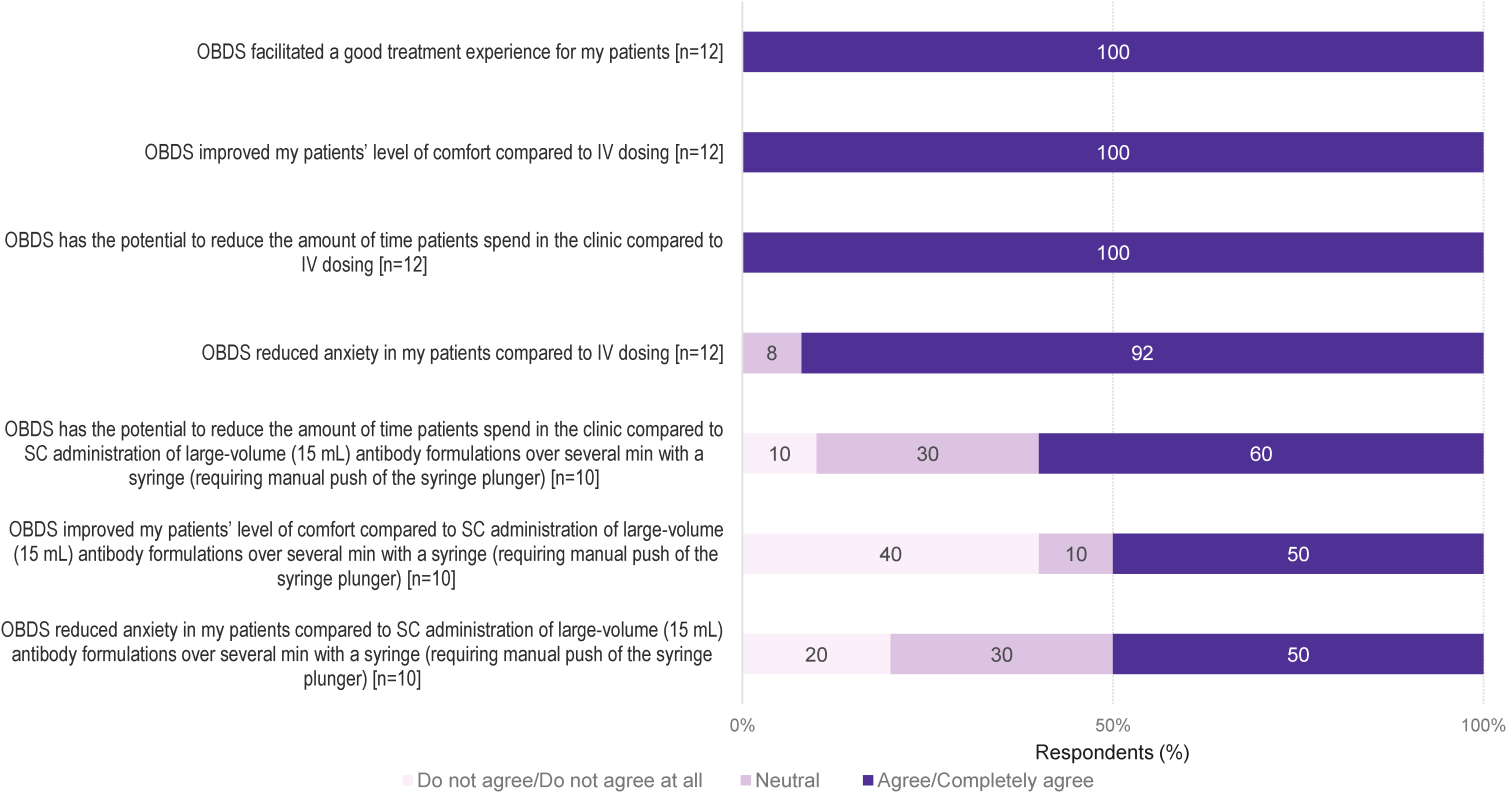
HCP observations of patients’ experiences with the OBDS compared with IV dosing and SC administration (by manual push) of large-volume (15 mL) antibody formulations with a syringe. A 5-point Likert scale was used to indicate the level of agreement, where 5 meant “I completely agree” and 1 meant “I do not agree at all”. 4 and 5 ratings were pooled as “agree/completely agree” and 2 and 1 ratings as “do not agree/do not agree at all”. HCP, healthcare professional; IV, intravenous; OBDS, on-body delivery system; SC, subcutaneous.

The nurses agreed that use of the OBDS facilitated a good treatment experience for their patients (100% of respondents; 12/12). Compared to IV dosing, it improved the patients’ level of comfort (100%; 12/12), could reduce the amount of time patients spent in the clinic (100%; 12/12), and decrease patient anxiety (92%; 11/12) (**Figure 2**). When compared to SC administration (by manual push) of large-volume (15 mL) antibody formulations with a syringe, the nurses agreed that use of the OBDS has the potential to reduce the amount of time patients spend in the clinic (60%; 6/10), improve the patient level of comfort (50%; 5/10), and decrease patient anxiety (50%; 5/10) (**Figure 2**).

Main advantages for patients receiving SC treatment via OBDS, as agreed upon by the nurses, included no needle visibility (100%, 12/12), short duration of treatment (100%; 12/12), a generally well-tolerated and painless SC injection via OBDS (83%; 10/12), and potential for at-home treatment (100%; 12/12) (**Table 3**). In the free-text responses, the nurses indicated time savings, less fear/stress, and greater comfort as advantages; 42% of the nurses (5/12) noted that automation of the injection process by the OBDS could reduce the risk of administration errors (**Supplementary Table S2**). Risk of allergy to the adhesive tape (50% of respondents; 6/12) and need to press and hold a button to pause the injection (17%, 2/12) were agreed upon by some of the nurses as potential limitations for patients (**Table 4**), although no allergy events nor interruptions were observed in 581 administrations of SC Isa via OBDS in the Phase 1b trial (18). In the free-text answers, a few of the nurses mentioned that the positioning required for SC administration may be more challenging for patients with compromised mobility (i.e. patients in a wheelchair). When asked about potential limitations for patients, 25% of the nurses (3/12) said “none”.

**Table 3:** Assessment of SC Isa treatment administration via OBDS using a 5-point scale: advantages for patients as perceived by HCPs.

Survey results	N	Mean rating	Median rating	Do not agree/do not agree at all (%)	Neutral (%)	Agree/completely agree (%)
No needle visibility	12	4.67	5.00	0	0	100
Short duration of treatment	12	4.58	5.00	0	0	100
Potential for at-home treatment	12	4.42	4.00	0	0	100
Generally, the OBDS is well tolerated and painless for patients	12	4.67	5.00	0	17	83
Increased convenience for patients, allowing standing position during the injection	12	2.67	2.50	50	33	17

A 5-point Likert scale was used to indicate the level of agreement, where 5 meant “I completely agree” and 1 meant “I do not agree at all”. 4 and 5 ratings were pooled as “agree/completely agree” and 2 and 1 ratings as “do not agree/do not agree at all”. HCP, healthcare professional; Isa, isatuximab; OBDS, on-body delivery system; SC, subcutaneous.

**Table 4:** Assessment of SC Isa treatment administration via OBDS using a 5-point scale: potential limitations for patients as perceived by HCPs.

Survey results	N	Mean rating	Median rating	Do not agree/do not agree at all (%)	Neutral (%)	Agree/completely agree (%)
Risk of allergy to the adhesive tape	12	3.50	3.50	17	33	50
Limitations in after-hours support for patients/caregivers during potential at-home administration	12	3.42	3.00	17	42	42
New caregivers to whom the patients are not accustomed to, if treatment is administered at home	12	3.08	3.00	25	33	42
Need to press and hold a button to pause the injection (active pause)	12	2.50	3.00	42	42	17

A 5-point Likert scale was used to indicate the level of agreement, where 5 meant “I completely agree” and 1 meant “I do not agree at all”. 4 and 5 ratings were pooled as “agree/completely agree” and 2 and 1 ratings as “do not agree/do not agree at all”. HCP, healthcare professional; Isa, isatuximab; OBDS, on-body delivery system; SC, subcutaneous.

### Monitoring of SC Isa administration via OBDS and confidence by treatment setting

**Figure 3:**
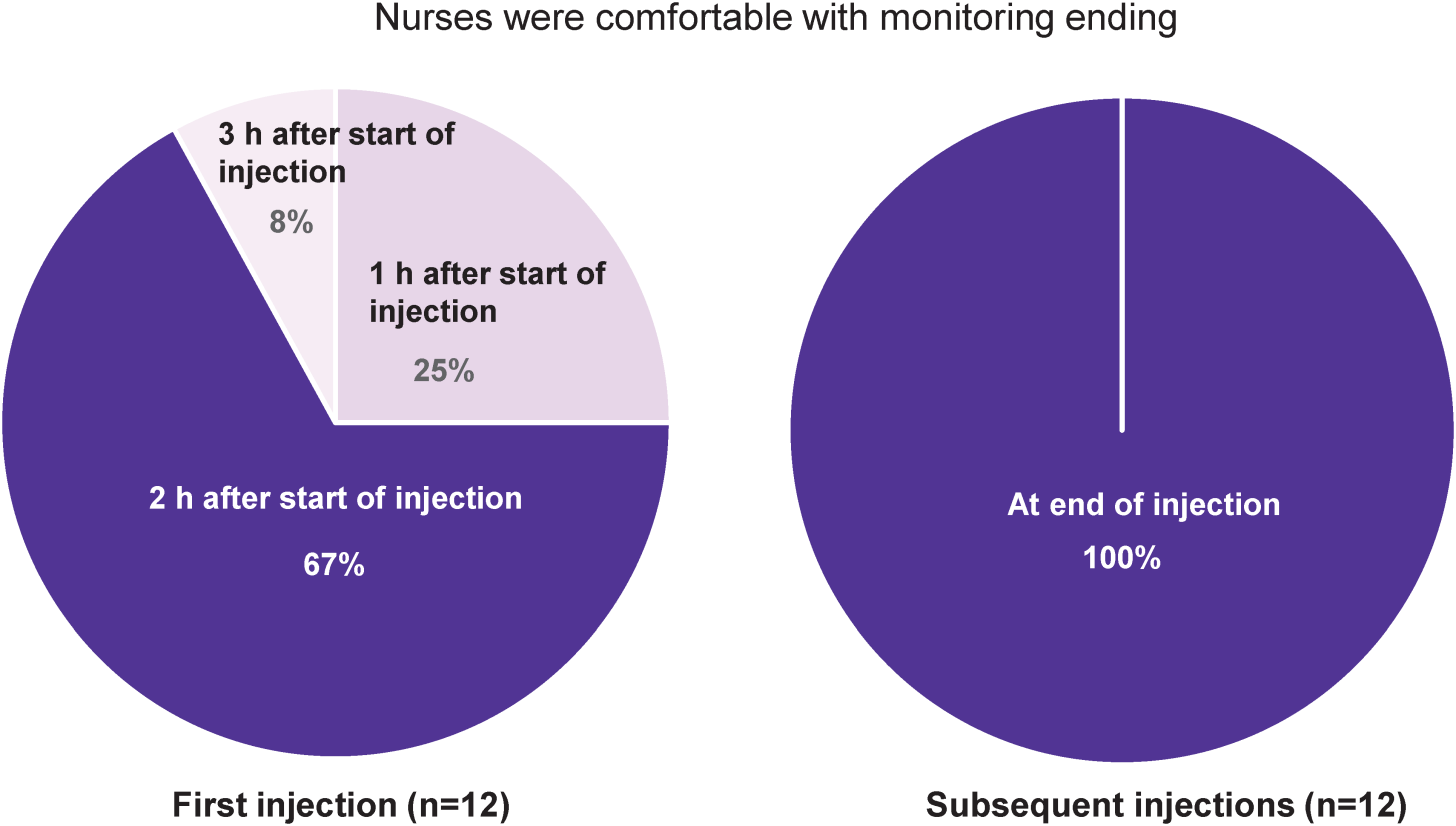
Monitoring of SC Isa administration via OBDS at first and subsequent injections. For first injection, 2 of the 3 nurses who reported being comfortable with monitoring ending 1 h after start of injection were from the GMMG-HD8 study, where monitoring was required for 1 h; the third nurse was from the Phase 1b study in which monitoring was required for up to 4 h after start of first injection. h, hour; Isa, isatuximab; OBDS, on-body delivery system; SC, subcutaneous.

Monitoring times for patients with MM receiving SC Isa therapy were implemented for first and subsequent injections according to each of the clinical study protocols for the Phase 1b and the Phase 3 GMMG-HD8/DSMM XIX trials (18, 19). In the survey, for the first administration, 25% of the nurses (3/12) reported that they were comfortable with monitoring ending 1 h after the start of the SC injection and 67% of them (8/12) with monitoring for 2 h from the start of the SC injection via OBDS (**Figure 3**). For subsequent administrations, all nurses (12/12) agreed they were comfortable with monitoring patients from start to end of the SC Isa injection via OBDS. In terms of clinical setting, all nurses expressed confidence in treatment delivery by an HCP in the clinic using the OBDS as well as in potential at-home treatment administration by an HCP via the OBDS, after a minimum of prior SC Isa cycles are given at the clinic.

## Discussion

We have reported here the results of a survey conducted among nurses with clinical experience in administering SC treatment with the anti-CD38 antibody Isa to patients with RRMM or NDMM, using an OBDS to help reduce time spent in the clinic and lighten the patient burden. The nurses invited to the survey had experience in multiple administration modalities for monoclonal antibodies (eg, IV infusion, SC administration by OBDS or manual push with a syringe), in addition to familiarity with clinical trial procedures, thus reflecting substantial clinical expertise and insight.

All the surveyed nurses expressed confidence in using the hands-free OBDS for SC Isa treatment as it was easy to learn and to administer, leading to improved efficiency with a low level of physical burden. As perceived by the nurses, use of the OBDS provided a good treatment experience for their patients with improved comfort, time savings, and decreased anxiety compared with IV dosing.

These findings of a positive patient experience with SC Isa administration via OBDS are in agreement with the results reported from a Patient Experience and Satisfaction Questionnaire at end of treatment (PESQ-EOT) in the Phase 1b study, which showed that most patients (87.5%) treated with SC Isa via OBDS were very satisfied or satisfied and very confident or confident in this novel treatment approach (23). In addition, the nurses’ assessment of SC Isa administration via OBDS as a time-saving procedure in this survey was in line with the reduction observed in a preliminary analysis of health resource utilization in the Phase 1b study in the time spent at the hospital for Isa administration via OBDS. As reported, median time from the patients’ arrival to departure decreased from 7 h at cycle 1 to 3.1 h at cycle 21, with a concomitant reduction in the time spent for patient monitoring (from 4 h at cycle 1 to 1.1 h at cycle 21) (23). For monitoring, the majority of the nurses on this survey were comfortable with monitoring for 2 h from start of injection for the first administration and all nurses in monitoring patients until the end of injection for subsequent administrations, based on their clinical experience with the OBDS and the clinical trial protocols. A short monitoring time would allow potential savings in terms of resources and lower the burden for the patients at the clinic.

Importantly, the time savings noted in this survey for patients receiving SC Isa may contribute to ensure adherence to treatment over time and thus result in optimal clinical outcomes, across the MM therapeutic spectrum. In this regard, the World Health Organization has indicated that both route and duration of treatment administration are therapy-related factors that can substantially influence adherence among patients receiving treatments on a long-term basis (24).

Although the small sample size represents a limitation in our analyses, the high degree of consistency frequently observed in the responses provided by the surveyed nurses, as well as their location in different countries and participation in different clinical trials, suggest that these results may have broad applicability. Results from further surveys may contribute to validate these initial findings in a larger group of HCPs with clinical experience in Isa SC administration using the OBDS.

As these expert nurses found that learning how to use and deliver antibody treatment with the OBDS was easy and preferable to standard, more cumbersome modalities, this novel OBDS approach may facilitate Isa treatment administration even more for nurses with less expertise in the delivery of antibody therapies, and thus contribute to reduce the risk of administration errors as noted in the survey. The lack of infusion/injection reactions and limited low-grade injection site reactions observed in the OBDS cohort of the Phase 1b study (1.7% of all injections) further underscore the safety and tolerability of this treatment approach (18). In addition, our findings are consistent with the results of a survey conducted among nurses with experience in SC administration of other monoclonal antibodies to cancer patients, who felt that use of an OBDS would improve efficiency in the clinic and decrease the nurse physical burden (25).

Future analyses in patients with NDMM or RRMM treated with SC Isa in large clinical trials may contribute to refine our understanding of specific steps as well as characteristics of the nurse and patient experiences with SC Isa treatment via OBDS. In this regard, the ongoing multinational, randomized, Phase 3 IRAKLIA/EFC15951 trial (NCT05405166) is evaluating efficacy and safety of SC Isa administered via OBDS compared to IV administration, both in combination with pomalidomide and dexamethasone, in patients with RRMM (20). This study will also include assessments of patient expectations, patient experience and satisfaction, as well as measures of health-related quality of life and healthcare resource utilization. Further data on patient-reported outcomes are expected from the ongoing, randomized, Phase 2 IZALCO study (NCT05704049) currently assessing SC Isa administered manually or via OBDS and from the Phase 2 SubQSA study (NCT06356571) of SC Isa administered via OBDS to patients with RRMM in combination with carfilzomib and dexamethasone (with weekly administration of carfilzomib in SubQSA) (21, 22).

In conclusion, the results from this survey show a high level of confidence among nurses as well as a preference for delivery of treatment with SC Isa using a hands-free OBDS compared with IV Isa administration, due to the ease of use, tolerability, and time savings achieved with OBDS injection. Our findings suggest applicability of the OBDS for convenient administration of SC Isa to patients with multiple myeloma in routine clinical practice.

## Data Availability

The original contributions presented in the study are included in the article/**Supplementary Material**. Further inquiries can be directed to the corresponding authors.
