# Supplementary material for: Subcutaneous administration of isatuximab in patients with multiple myeloma by an on-body delivery system: results of a nurse survey
Source: Front Oncol. 2025 Jun 18;15:1547108. doi: 10.3389/fonc.2025.1547108 (PMC12213733; doi:10.3389/fonc.2025.1547108)
Supplement: Supplementary file 1 [file DataSheet1.pdf]

SUPPLEMENTARY INFORMATION

Subcutaneous administration of isatuximab in patients with multiple myeloma by an on-body delivery system: results of a nurse survey

Nuria Sánchez Avello, Paula Calvo Pajares, Paul Cordero, Florence Suzan, Connie Barlas

Supplementary Figure S1 On-body delivery system (OBDS ST) wearable injector*.

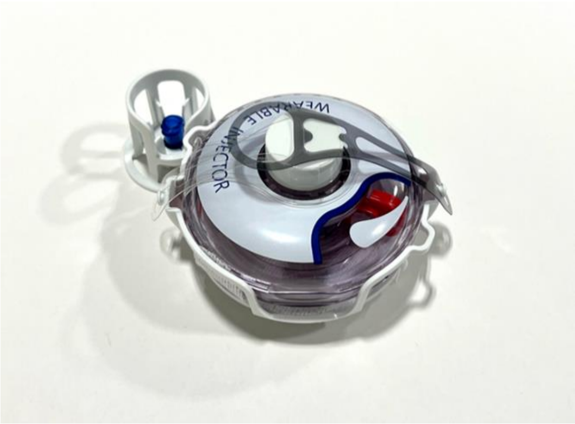

*CAUTION – Investigational device. Limited by Federal (or United States) law to investigational use. ST, device filling by syringe transfer.

Supplementary Table S1 Assessment of SC Isa treatment administration via OBDS: advantages for HCPs – unaided, free-text answers.

Question/request in the survey: "Please enter any advantages of the on-body delivery system (OBDS) for HCPs in the clinic, into the free-text box below."

Respondent	Free-text Answers
1	▪ Convenient, functional, and fast.
2	▪ Easy to handle and there is no danger of exposure to the product (no risk of aerosol particles or injection with a needle).<br>▪ Saves time.<br>▪ Simplified asepsis.
3	▪ Ease of handling and use.
4	▪ Easy to use.<br>▪ Administrator does not have to sit and manually push the medication.<br>▪ The device delivers at a more consistent rate.
5	▪ Optimizing physical health care resources.<br>▪ Ease of administration.<br>▪ Less time spent in the outpatient area for patients.<br>▪ Better administration in the best possible timeframe compared to manual.
6	▪ Decreased nursing burden.<br>▪ Patients spend less time in the day hospital.
7	▪ Decrease manual effort in administration (automatic system).<br>▪ Optimization of physical resources in the administration area (less time spent in the chair), shorter time spent in the outpatient area.<br>▪ Better tolerability.
8	▪ The on-body delivery system (OBDS) is first of all a much less invasive route of administration than the venous route. Our patients have often received several lines of treatments, therefore with very precarious venous capital.<br>▪ In addition, it is a much less painful administration method for these chronic patients.<br>▪ Finally, the closed system is an additional safety means for us nurses when handling products.
9	▪ Simplifies administration of treatment.<br>▪ Accuracy of the drug administration compared to the manual.<br>▪ Optimizes physical resources for help.
10	▪ Saves time.<br>▪ No pain in our hand that pushes the syringe by 15 ml

11	▪ Time saving.<br>▪ Little pain for the patient during the injection.<br>▪ Easy to use, error minimization.<br>▪ Very low risk of injury
12	▪ Time-saving.<br>▪ Space-saving (bed occupancy).<br>▪ Easy to use.

HCP, healthcare professional; Isa, isatuximab; OBDS, on-body delivery system; SC, subcutaneous.

Supplementary Table S2 Assessment of SC Isa treatment administration via OBDS: advantages for patients as perceived by HCPs – unaided, free-text answers.

Question/request in the survey: "Please enter any advantages of the on-body delivery system (OBDS) for patients into the free-text box below."

Respondent	Free-text Answers
1	• Reduced time in hospital vs intravenous injection.
2	• Patients are curious about administration. It is a simple to use system that could be performed by the patients themselves.<br>• Less anxiety about the needle too.
3	• Non-painful during application.<br>• No skin reaction with the glue of the device.
4	• Less user/administration error.<br>• More privacy, as you do not have a clinician seated right next to you administering the injection.<br>• Quicker and more predictable.
5	• Better administration time.<br>• Less likelihood of manual mismanagement.<br>• Decreased administration time compared to intravenous.
6	• Reduced administration time.<br>• Decrease in likelihood of administration errors
7	• Automatic administration, does not depend on the nurse's manual effort.<br>• Shorter time, less time than if it were introvenous.<br>• Decreased administration errors (greater safety).
8	• Faster, more comfortable administration method.<br>• Patients remember a positive experience and return without fear for other sessions.
9	• Reduction administration time compared to intravenous.<br>• Reduces the risk of making administration errors.<br>• Shorter administration time.
10	• Speed of injection upon receipt of the product with premedication (IV 1h15 plus premedication).<br>• Saves time and is less stressful when patient is difficult to inject.
11	• Less fear.<br>• The needle is not visible.<br>• Less pain.<br>• Less time required compared with intravenous administration.

12	• Time-saving, shorter stays in the hospital (lower risk of infection from other patients).<br>• Less psychological stress.<br>• No port necessary.<br>• Fewer reactions to the antibodies.

HCP, healthcare professional; Isa, isatuximab; OBDS, on-body delivery system; SC, subcutaneous.
